# Coronary ^18^F-Fluoride Uptake and Progression of Coronary Artery Calcification

**DOI:** 10.1161/CIRCIMAGING.120.011438

**Published:** 2020-12-15

**Authors:** Mhairi K. Doris, Mohammed N. Meah, Alastair J. Moss, Jack P.M. Andrews, Rong Bing, Rebecca Gillen, Nick Weir, Maaz Syed, Marwa Daghem, Anoop Shah, Michelle C. Williams, Edwin J.R. van Beek, Laura Forsyth, Damini Dey, Piotr J. Slomka, Marc R. Dweck, David E. Newby, Philip D. Adamson

**Affiliations:** 1 British Heart Foundation Centre for Cardiovascular Science (M.K.D., M.N.M., A.J.M., J.P.M.A., R.B., M.S., M.D., A.S., M.C.W., E.J.R.v.B., M.R.D., D.E.N., P.D.A.), University of Edinburgh, United Kingdom.; 2 Edinburgh Clinical Trials Unit (L.F.), University of Edinburgh, United Kingdom.; 3 Edinburgh Imaging, Queen’s Medical Research Institute University of Edinburgh, Edinburgh, United Kingdom (Rebecca Gillen, Nick Weir, Michelle C Williams, Edwin JR van Beek, David E Newby).; 4 Division of Nuclear Medicine, Department of Imaging, Medicine, and Biomedical Sciences, Cedars-Sinai Medical Center, Los Angeles, CA (D.D., P.J.S.).; 5 Christchurch Heart Institute, University of Otago, Christchurch, NZ (P.D.A.).

**Keywords:** atherosclerosis, calcium, coronary angiography, positron emission tomography, risk factors

## Abstract

**Background:**

Positron emission tomography (PET) using ^18^F-sodium fluoride (^18^F-fluoride) to detect microcalcification may provide insight into disease activity in coronary atherosclerosis. This study aimed to investigate the relationship between ^18^F-fluoride uptake and progression of coronary calcification in patients with clinically stable coronary artery disease.

**Methods:**

Patients with established multivessel coronary atherosclerosis underwent ^18^F-fluoride PET-computed tomography angiography and computed tomography calcium scoring, with repeat computed tomography angiography and calcium scoring at one year. Coronary PET uptake was analyzed qualitatively and semiquantitatively in diseased vessels by measuring maximum tissue-to-background ratio. Coronary calcification was quantified by measuring calcium score, mass, and volume.

**Results:**

In a total of 183 participants (median age 66 years, 80% male), 116 (63%) patients had increased ^18^F-fluoride uptake in at least one vessel. Individuals with increased ^18^F-fluoride uptake demonstrated more rapid progression of calcification compared with those without uptake (change in calcium score, 97 [39–166] versus 35 [7–93] AU; *P*<0.0001). Indeed, the calcium score only increased in coronary segments with ^18^F-fluoride uptake (from 95 [30–209] to 148 [61–289] AU; *P*<0.001) and remained unchanged in segments without ^18^F-fluoride uptake (from 46 [16–113] to 49 [20–115] AU; *P*=0.329). Baseline coronary ^18^F-fluoride maximum tissue-to-background ratio correlated with 1-year change in calcium score, calcium volume, and calcium mass (Spearman ρ=0.37, 0.38, and 0.46, respectively; *P*<0.0001 for all). At the segmental level, baseline ^18^F-fluoride activity was an independent predictor of calcium score at 12 months (*P*<0.001). However, at the patient level, this was not independent of age, sex, and baseline calcium score (*P*=0.50).

**Conclusions:**

Coronary ^18^F-fluoride uptake identifies both patients and individual coronary segments with more rapid progression of coronary calcification, providing important insights into disease activity within the coronary circulation. At the individual patient level, total calcium score remains an important marker of disease burden and progression.

**Registration:**

URL: https://www.clinicaltrials.gov; Unique identifier: NCT02110303.

## Abstract 2

Supplemental Digital Content is available in the text.

**CLINICAL PERSPECTIVE**

Positron Emission Tomography using the radiotracer ^18^F-fluoride can identify microcalcification and high-risk plaque. In this study, the relationship between ^18^F-fluoride activity and the progression of coronary calcification was investigated in patients with clinically stable multivessel coronary artery disease. The results demonstrated more rapid progression of coronary calcification in patients with increased ^18^F-fluoride activity at baseline. In individual lesions, baseline ^18^F-fluoride activity was an independent predictor of progression of calcification at one year. At the patient level, baseline calcium burden remained a more powerful predictor of calcium score at one year. These findings suggest that ^18^F-fluoride activity can provide an insight into disease activity in coronary atherosclerosis. By providing early evidence of calcification activity, ^18^F-fluoride uptake may be useful in clinical trials of novel therapies designed to modify disease progression. In the clinical setting, identifying patients with increased disease activity at risk of more rapid disease progression may enable targeting of intensified therapy.

**See Article by Chareonthaitawee and Hyafil**

Atherosclerotic calcification is a complex pathophysiological process central to the development of advanced coronary artery plaques. Macroscopic coronary artery calcification can be quantified using computed tomography, serving as an important surrogate measure of atherosclerotic plaque burden and providing powerful prognostic information beyond traditional risk factors.^1–3^ Furthermore, progression of coronary macrocalcification is associated with an increased risk of adverse cardiovascular events and all-cause mortality.^4–6^

The positron-emitting radiotracer ^18^F-sodium fluoride (^18^F-fluoride), commonly used to detect pathological metabolic activity in the skeletal system,^7,8^ can provide important insights into calcification activity within the cardiovascular system. ^18^F-fluoride binds to hydroxyapatite in proportion to the surface area of exposed crystal, such that it binds preferentially to areas of newly developing microcalcification where the surface area is many fold higher than large macroscopic deposits.^9^ Microcalcification is a key healing response in atherosclerotic plaque,^9^ closely associated with necrotic inflammation and high-risk disease, but ultimately leading to downstream coronary macrocalcification and plaque stabilization. ^18^F-fluoride positron emission tomography (PET)-CT, therefore, has the potential to act as a marker of disease activity in the coronary vasculature by targeting a biologically important stage of the disease.^10^ Indeed, ex vivo studies have confirmed the exquisite sensitivity of ^18^F-fluoride for early microcalcific deposits and its association with high-risk plaque features including macrophage infiltration and necrosis in atherosclerosis.^11–13^

Based on these principles, we hypothesized that coronary ^18^F-fluoride PET-CT should predict the future progression of coronary macrocalcification detected on CT. We, therefore, prospectively investigated the relationship between in vivo coronary ^18^F-fluoride uptake and the progression of coronary arterial calcification in patients with clinically stable coronary artery disease.

## Methods

### Study Design

This study is a prespecified analysis of an investigator-initiated double-blind randomized controlled trial conducted at a single centre in Edinburgh, UK.^11^ The study was approved by the local institutional review board, the Scottish Research Ethics Committee (REC reference: 14/SS/0089), Medicines and Healthcare products Regulatory Agency, and the United Kingdom Administration of Radiation Substances Advisory Committee. The study was performed in accordance with the Declaration of Helsinki and all patients provided written informed consent before any study procedures. The data that support the findings of this study are available from the corresponding author upon reasonable request.

### Study Population

Patients with clinically stable multivessel coronary artery disease were recruited prospectively from the Edinburgh Heart Centre, United Kingdom, between March 2015 and March 2017. Patients were included if aged over 40 years and with evidence of angiographically proven multivessel coronary artery disease, defined as at least 2 major epicardial vessels with any combination of either (1) >50% luminal stenosis or (2) previous revascularization (percutaneous coronary intervention or coronary artery bypass graft surgery). Patients were excluded in the event of coronary revascularization within the preceding 3 months or acute coronary syndrome within the previous 12 months (Table I in the Data Supplement). Patients were randomized (1:1) to receive ticagrelor or placebo in addition to aspirin therapy, and the primary goal of the original study was to determine whether ticagrelor therapy, in addition to aspirin, reduces high-sensitivity troponin I concentration in participants with high-risk coronary plaque. The primary study results have been previously reported.^14^

### Study Procedures

All participants underwent a baseline clinical assessment and combined ^18^F-fluoride PET-CT scanning with the acquisition of a contrast-enhanced CT coronary angiogram and noncontrast CT for calcium scoring. Before scanning, participants with a resting heart rate >65 beats/min were administered oral β-blockade (50–100 mg metoprolol) unless contraindicated. All participants were administered a target dose of 250 MBq intravenous ^18^F-fluoride and rested in a quiet environment. Sixty minutes following injection, PET acquisition was performed on a hybrid PET-CT scanner (64-multidetector Biograph mCT, Siemens Medical Systems, Erlangen, Germany). Attenuation correction CT scans were performed before the acquisition of ECG-gated list-mode PET data using a single 30-minute bed position centred on the heart. Finally, an ECG-gated coronary CT angiogram was performed in mid-diastole during held expiration following sublingual glyceryl trinitrate. Repeat coronary CT angiography and calcium scoring were performed using the same imaging protocol and on the same scanner at one year.

### Image Analysis

#### Calcium Scoring

Noncontrast CT images for calcium scoring were reconstructed in the axial plane with 3 mm slice width and 1.5 mm increment. Coronary calcium was quantified on both a per-participant and per-segment level by an experienced observer using dedicated software (Vitrea Advanced, Toshiba Systems). Calcification was quantified as calcium score (AU), calcium volume (mm^3^) and calcium mass (mg). Calcium score was derived using the Agatston method.^15^ To calculate calcium mass, a calibration factor was derived using a phantom to calculate equivalent water diameter, adjusted for body mass index and lateral diameter and applied at a specified X-ray tube voltage (Table II in the Data Supplement).^16^ Coronary stents were excluded from the per-patient analysis by only including calcium proximal or distal to the border of the stented segment. For the per-segment analysis, only segments without stenting were selected as representative positive and negative segments.

#### Coronary CT Angiography Image Analysis

Visual assessment of CT-defined high-risk plaque characteristics was performed by trained observers. The presence or absence of 5 plaque characteristics (positive remodeling, low attenuation plaque, the napkin ring sign, spotty calcification, and punctate calcification) was documented on a segmental basis using a 15-segment model.^17^ Positive remodeling was defined as a vessel diameter of 10% greater than a reference segment proximal to the plaque.^18^ Low attenuation plaque was defined as a focal area of plaque with an attenuation density of <30 Hounsfield units.^19^ Spotty calcification was defined as focal areas of arterial calcification with a maximum diameter <3 mm. The napkin ring sign was defined as a central area of low attenuation plaque surrounded by a rim of high attenuation, as described previously.^20^

Coronary artery plaque quantification was performed on all nonstented segments with visible atherosclerotic plaque by a trained observer using semi-automated software (Autoplaque, Cedars Sinai Medical Center, Los Angeles). Plaque components including remodeling index, calcified plaque, noncalcified plaque and low attenuation plaque volume were quantified using scan-specific thresholds referencing blood pool attenuation from a circular region of interest created in the proximal ascending aorta. Manual adjustments were made as required. This technique has previously demonstrated excellent intraobserver, inter-observer, and scan-rescan reproducibility.^21,22^

#### PET Image Analysis

PET images were reconstructed in diastole (50%–75% of the R-R interval, 2 iterations, 21 subsets, 5 mm gaussian smoothing, Siemens Ultra-HD algorithm) and fused with contrast-enhanced CT coronary angiography. Images were co-registered on 3 anatomic planes and qualitative and semi-quantitative analysis of coronary ^18^F-fluoride uptake was performed by experienced observers. Coronary ^18^F-fluoride PET-CT analysis and reproducibility have been reported previously.^16^ In brief, visual assessment for increased coronary ^18^F-fluoride uptake was performed on both a per-participant and per-segment level and deemed positive if there was focal radiotracer accumulation which co-localized to an atherosclerotic plaque on coronary CT angiography and followed the course of the coronary artery >5 mm in axial, sagittal, and orthogonal views. Where visual uptake was identified, semi-quantitative analysis was performed by drawing 2-dimensional regions of interest on the axial images and quantifying the maximum standardized uptake value for that lesion. In each participant, maximum standardized uptake value was also quantified in a proximal coronary atherosclerotic plaque without visual evidence of radiotracer localization as a reference. Background blood pool activity was quantified by measuring activity in the right atrium within 2 cm radius regions of interest on three consecutive slices. Maximum tissue-to-background ratio (TBRmax) was calculated by dividing the plaque maximum standardized uptake value by the mean average standardized uptake value in the right atrium. Coronary plaques were considered PET-positive in the presence of focal radiotracer uptake localized to a plaque with a TBRmax >1.25, as described previously.^11^ For the purpose of the per-segment analysis, participants were only included if there were both a PET-positive and PET-negative plaque which were not stented (Figure I in the Data Supplement). Participants in which the only PET-positive or PET-negative plaque was stented were excluded from per-segment analysis. Image analysis was performed using an OsiriX workstation (OsiriX version 3.5.1 64-bit; OsiriX Imaging Software, Geneva, Switzerland).

### Statistical Analysis

Categorical variables are reported as number (%) and continuous variables as mean±SD for parametric or median (interquartile range) for nonparametric data. Continuous unpaired variables were compared using Student *t* test or the Mann-Whitney *U* test where appropriate and paired variables compared using Student *t* test or the Wilcoxon matched-pairs signed-ranks test, dependent on normality. Categorical variables were compared using χ^2^ tests. Logarithmic transformation (Ln[x+1]) was used to achieve normality of continuous variables. Two-tailed Pearson correlation analysis was performed to investigate the relationship between continuous variables where normally distributed. Nonparametric continuous variables were compared using Spearman rank correlation. To investigate the relationship between ^18^F-fluoride activity and progression of coronary calcification at the patient level, linear regression was used with log transformation of the dependent variable. To investigate at the per-segment level, a linear mixed model was used with the participant as a random effect to adjust for repeated measurements within individuals. Using calcium mass at 12 months as the dependent variable, ANOVA was then used to compare 2 mixed models; model one using calcium mass at baseline, and model 2 using baseline calcium and segmental TBR as independent variables. Statistical analysis was undertaken using IBM SPSS Statistics 23 and R version 3.5.0 (R Foundation for Statistical Computing, Vienna, Austria). Statistical significance was considered as a 2-sided *P*<0.05.

## Results

A total of 185 participants underwent combined PET-CT angiography and CT calcium scoring at baseline and follow-up (mean age 65±8, 80% male; Figure II in the Data Supplement). For the purposes of this analysis, we excluded participants who underwent coronary revascularization during the course of the trial with implantation of a coronary stent within a previously unstented coronary segment (n=2). Coronary revascularization decisions were made by the attending clinician independent of the research study team or knowledge of the PET-CT scan findings. Of the participants who underwent percutaneous coronary intervention, both had evidence of increased ^18^F-fluoride activity. One participant underwent stenting to the left anterior descending artery and left circumflex artery, and the other underwent stenting to the left anterior descending artery. All 3 lesions showed obstructive stenosis on the baseline scan (70%–99% stenosis) and 2 of 3 had high-risk plaque features (positive remodeling and spotty calcification). In the remaining eligible participants (n=183), there was a high prevalence of cardiovascular risk factors, of which 69% (n=126) had a history of acute coronary syndrome, 81% (n=148) of participants had previous percutaneous coronary intervention, and the majority 96% (n=175) were on statin therapy (Table 1).

**Table 1.**
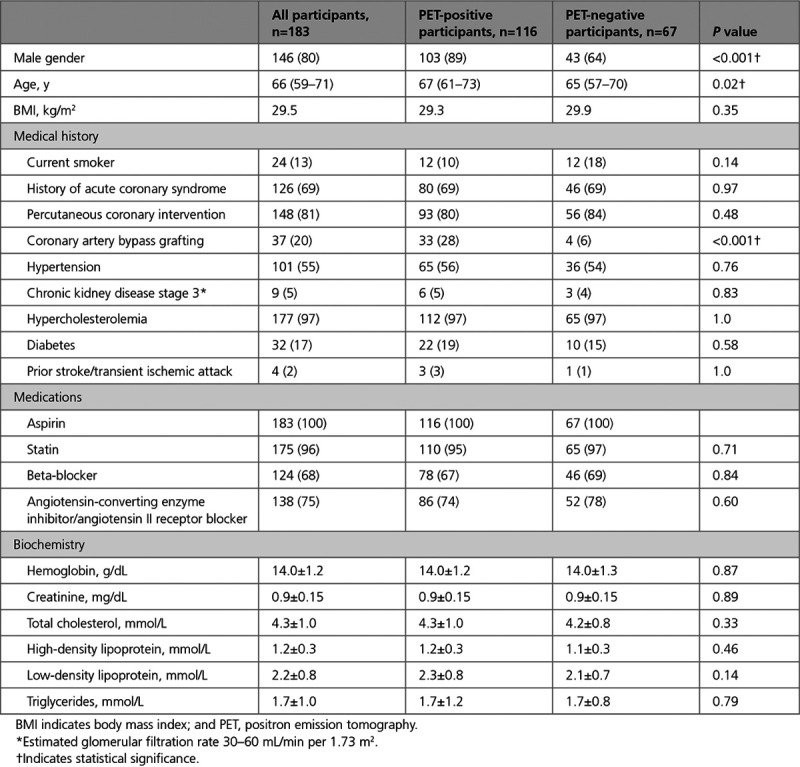
Baseline Characteristics

Of the 183 participants included in this analysis, 63% (n=116) patients had evidence of increased ^18^F-fluoride uptake in at least one vessel (*PET*-positive). The PET-positive cohort was predominantly male (89%, n=103% versus 64%, n=43; *P*<0.001), with a higher prevalence of prior coronary artery bypass graft surgery (28%, n=33% versus 6%, n=4; *P*<0.001).

### Baseline ^18^F-Fluoride Activity and Per-Participant Calcium Burden

**Figure 1.**
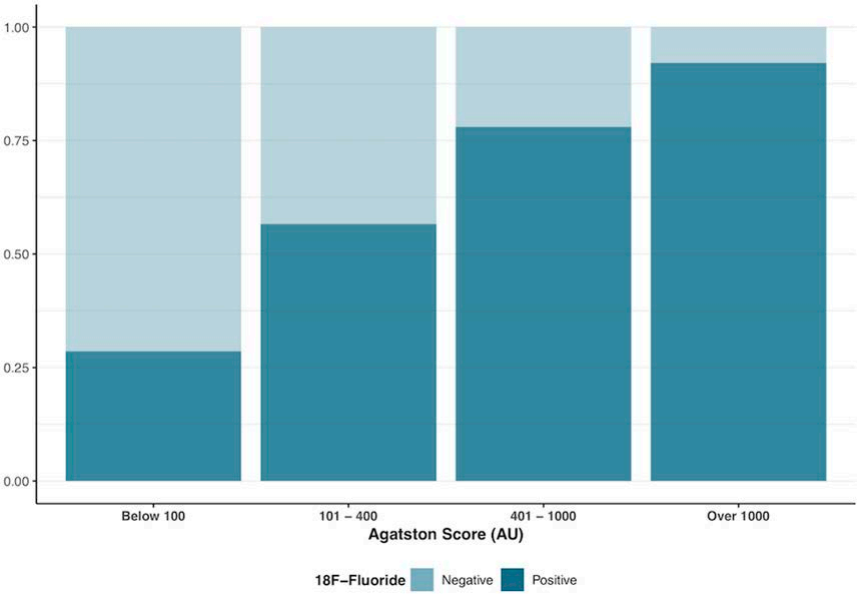
**The proportion of ^18^F-fluoride positive and negative participants with increasing baseline total calcium score.** Increased overall disease burden is associated with an increased frequency of ^18^F-fluoride activity.

Participants with increased coronary ^18^F-fluoride uptake had higher baseline calcium scores (524 [242–1091] versus 136 [55–361] AU; *P*<0.001), higher calcium mass (99 [46–212] versus 24 [11–69] mg; *P*<0.0001), and higher calcium volume (491 [247–984] versus 131 [64–343] mm^3^; *P*<0.0001) compared with those without increased ^18^F-fluoride uptake (Table 2). The proportion of PET-positive participants rose with increasing baseline calcium score (Figure 1). Thirty-eight participants had a total calcium score >1000 Agatston units at baseline and, of these, 35 (92%) had evidence of increased ^18^F-fluoride uptake in at least one vessel.

**Table 2.**
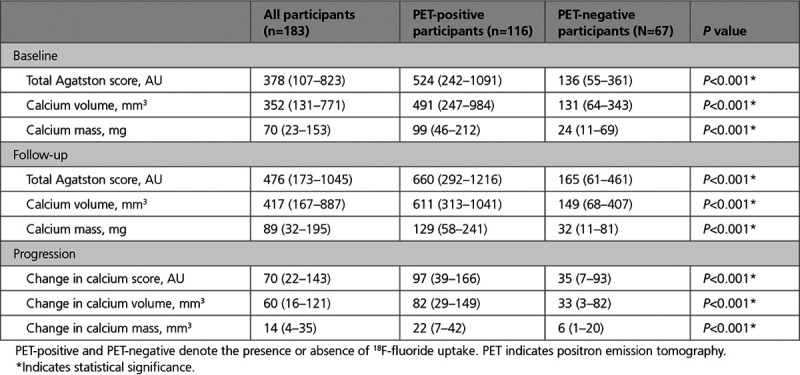
Progression of Calcification in Participants With Evidence of Increased ^18^F-Fluoride Uptake Compared With Those Without Uptake

### ^18^F-Fluoride Uptake and Plaque Characteristics

Sixty (52%) of the PET-positive participants and 37 (55%) of the PET-negative participants had at least one high-risk plaque feature on CT coronary angiography. There was no difference in the median number of high-risk plaque features per-patient in PET-positive (1.0 [0.0–2.3]) compared with PET-negative (1.0 [0.0–3.0]) participants (Table III in the Data Supplement). Nine participants were excluded from quantitative plaque analysis due to suboptimal image quality. On quantitative plaque analysis in the remaining 174 participants, total plaque volume, noncalcified plaque volume, and calcified plaque volume were higher in PET-positive participants at baseline and at 1-year follow-up (Table 3).

**Table 3.**
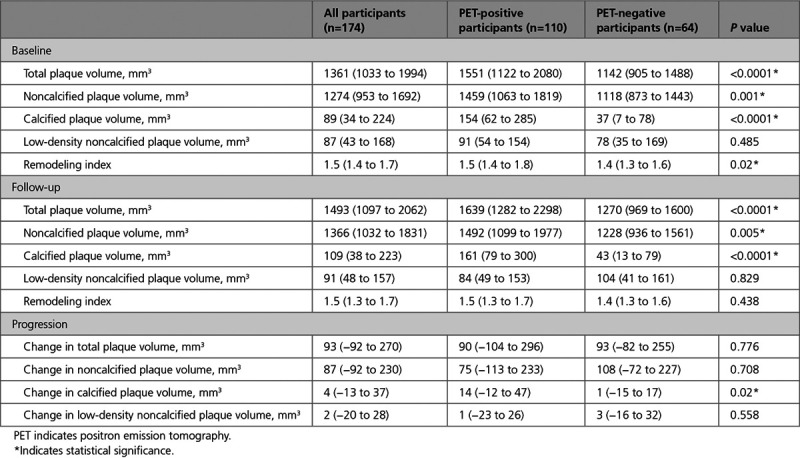
Quantitative Plaque Features in PET-Positive and PET-Negative Participants

### ^18^F-Fluoride Uptake and Progression of Calcification on a Per-Patient Level

The median increase in calcium score, calcium mass, and calcium volume were higher in PET-positive compared with PET-negative participants (*P*<0.001 for all; Table 2). Change in total calcified plaque volume on CT coronary angiography was also higher in PET-positive participants. There was no difference in change in noncalcified plaque, low-density plaque, or total plaque volume (Table 3). Per-patient TBRmax correlated with change in calcium score (Spearman ρ=0.37), calcium mass (ρ=0.46), and calcium volume (ρ=0.38; *P*<0.001 for all; Table 4). There was a weak positive correlation between baseline TBRmax and change in total calcified plaque volume (Spearman’s ρ, 0.165; *P*=0.029; Table 4).

**Table 4.**
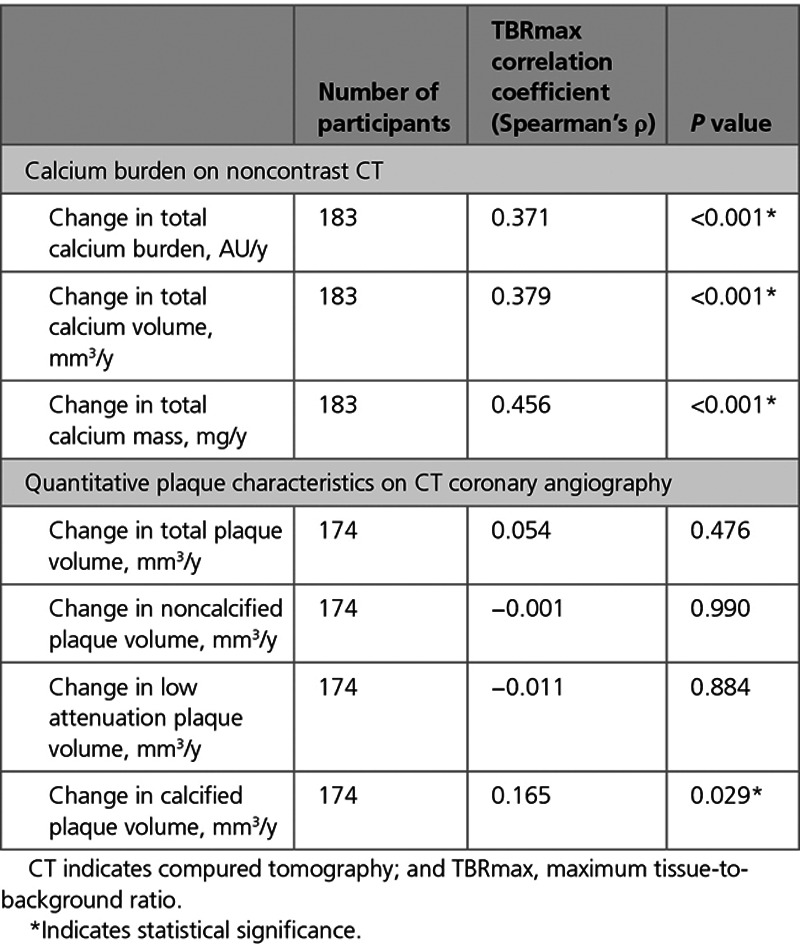
Correlation Between Baseline TBRmax and Progression of Calcification

After adjusting for age, sex, and baseline calcium score, baseline ^18^F-fluoride activity was not an independent predictor of calcium score at 12 months when investigated at the patient level (*P*=0.50).

### Per-Segment ^18^F-Fluoride Activity and Progression of Calcification

**Figure 2.**
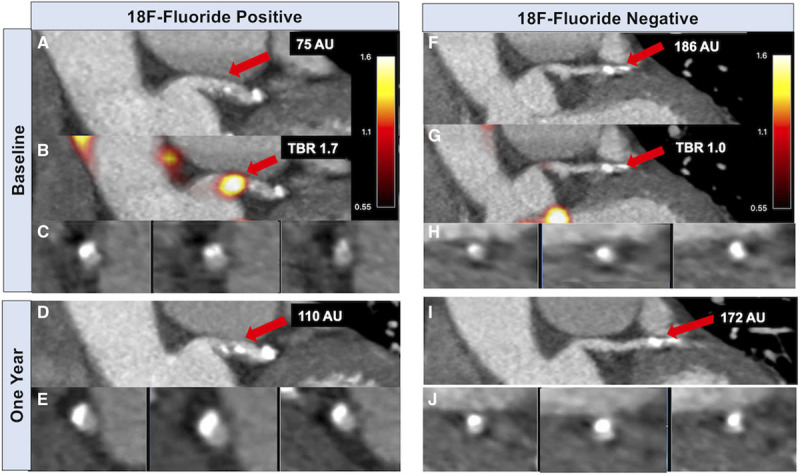
**^18^F** **-fluoride activity predicts progression of coronary arterial calcification.** In an ^18^F-fluoride positron emission tomography (PET) positive lesion (**A**–**E**), contrast-enhanced computed tomography (CT) coronary angiography (**A**) and fused PET-CT (**B**) demonstrate ^18^F-fluoride uptake in the left main stem (LMS) at baseline overlying a mixed plaque shown on coronal view (**A**) and cross-section (**C**). Repeat CT coronary angiography at one year demonstrates progression of calcification in this segment with a higher calcium score (**D**) and dense calcium visible on cross-section (**E**). Calcium score 75 AU at baseline, maximum tissue-to-background ratio (TBR_max_) 1.7, calcium score 110 AU at one year. In the same patient, a calcified plaque in a proximal obtuse marginal branch without evidence of increased ^18^F-fluoride activity is shown on coronal (**F** and **G**) and cross-section views (**H**). This plaque does not demonstrate progression in calcium score at one year (**I** and **J**). Calcium score 186 AU at baseline, TBR_max_ 1.0, calcium score 172 AU at one year.

Calcification in individual coronary segments with evidence of ^18^F-fluoride uptake was compared with a proximal reference segment with atherosclerotic plaque but without increased ^18^F-fluoride uptake within the same individual (Figure 2). Six participants, in whom the only PET-positive lesion or only PET-negative lesion was stented, were excluded from analysis. Among the remaining PET-positive participants (n=110), lesions with ^18^F-fluoride uptake had a higher calcium score, calcium mass, and volume at baseline than PET-negative reference lesions in the same individuals (*P*<0.001 for all; Table 5). There was no difference in baseline average calcium density or change in average calcium density between PET-positive and PET-negative lesions (Supplemental Table IV in the Data Supplement).

**Table 5.**
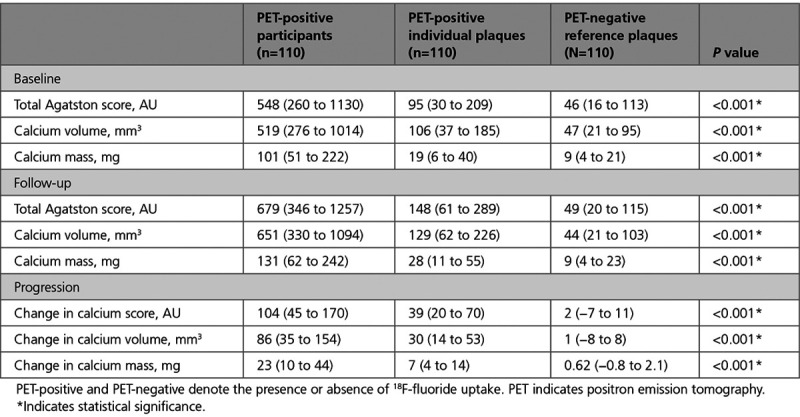
Progression of Calcification at a Segmental Level in Coronary Arterial Segments With and Without Increased ^18^F-Fluoride Uptake in PET Positive Participants

**Figure 3.**
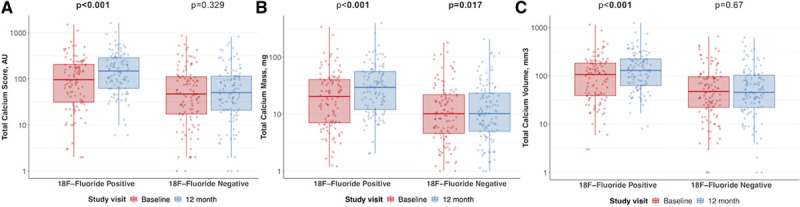
**The relationship between baseline ^18^F** **-fluoride activity and coronary calcification at one year.** **A**–**C**, Display the change in calcium score (**A**), mass (**B**), and volume (**C**) in ^18^F-fluoride positive vs negative lesions over 12 months. Median and interquartile range displayed.

**Figure 4.**
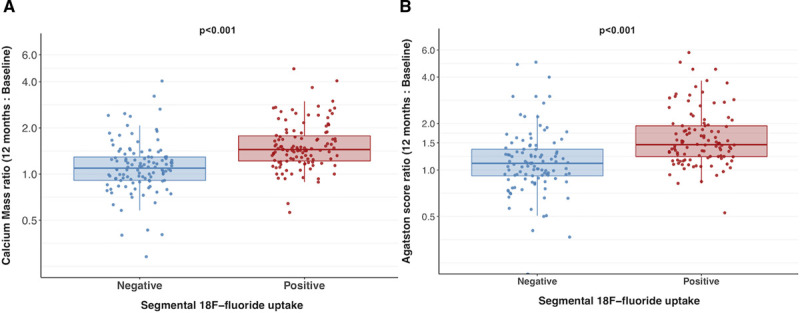
**Ratio of calcification at follow-up to baseline**. Ratio of follow-up total calcium mass (**A**) and Agatston score (**B**) to baseline in individual positron emission tomography (PET)-positive and PET-negative segments in participants with at least one PET-positive lesion.

In PET-positive segments, there was an increase in calcium score (from 95 [30–209] to 148 [61–289] AU), calcium mass (from 19 [6–40] to 28 [11–55] mg), and calcium volume (from 106 [37–185] to 129 [62–226] mm^3^; *P*<0.001 for all). In PET-negative reference segments, there was no change in calcium score (from 46 [16–113] to 49 [20–115] AU; *P*=0.329) and calcium volume (from 46–44 mm^3^, *P*=0.666), although there was a small increase in calcium mass (from 9.1–9.2 mg; *P*=0.017; Figure 3). Similarly, changes in calcification were greater in PET-positive plaques compared with PET-negative plaques for the calcium score (39 [20–70] versus 2 [−7 to 11] AU), calcium mass (7 [4–14] versus 0.6 [−0.8 to 2.1] mg) and calcium volume (30 [14–53] versus 1 [−8 to 8] mm^3^; *P*<0.001 for all). When segmental calcium mass and calcium score were measured as a ratio of follow-up to baseline, the calcium mass and calcium score ratio were each higher in PET-positive segments (Figure 4).

When the relationship between ^18^F-fluoride activity and progression of calcification was investigated at the segmental level, baseline TBRmax was an independent predictor of 12-month calcium mass when adjusting for baseline calcium mass (beta 0.67 SE 0.06), Agatston score (beta 0.60 SE 0.09) or volume (beta 0.52 SE 0.07), and the repeated within individual measurements (*P*<0.001 for all).

## Discussion

In this prespecified sub-study of a randomized controlled trial, we have demonstrated that increased ^18^F-fluoride uptake is associated with more rapid progression of coronary atherosclerotic calcification. This finding was consistent across a range of measures of calcification and whether this was considered on a per-patient or per-segment basis. However, when considering total calcification burden at the patient level, baseline calcium score remained a powerful predictor of calcification progression. These findings nonetheless support ^18^F-fluoride PET as a marker of increased disease activity and active mineralization within coronary atherosclerotic plaques.

Calcification plays a complex role in the development and progression of atherosclerosis. Hydroxyapatite deposition is heralded by intense inflammation, cell death and necrosis which precipitate the formation of calcifying matrix vesicles and lead to the earliest microscopic deposits of calcium which are not visible on conventional anatomic imaging modalities.^10,12,23^ As well as reflecting the proinflammatory environment, microcalcification may also be directly implicated in plaque rupture as a consequence of destabilization of the plaque’s fibrous cap.^24^ As microcalcific deposits coalesce to form larger macroscopic structures, plaque stability improves—a feature reflected by the observation that heavily calcified plaques are less prone to rupture or precipitate acute coronary events.^25,26^ Thus, while advanced macrocalcification may be considered a marker of plaque stability and inert disease, the earlier stages of calcium deposition are associated with increased inflammation and plaque vulnerability.^23,27^

The precise natural history of calcification activity remains poorly understood. Indeed, the process of calcification does not progress in a linear manner and, while the earliest microcalcific deposits are associated with intense inflammation,^10^ it is likely that active calcification may accelerate during the process of plaque healing. This observation is concordant with prior randomized trials that have observed a procalcific effect of statins due to phenotypic transformation of low attenuation material to calcified plaque.^24^ While computed tomography can provide quantitative measures of total plaque burden, it does not provide insight into the underlying biological disease process or disease activity, hence the rationale for using targeted radiotracers.

^18^F-fluoride preferentially binds developing microcalcification,^24^ with uptake, therefore, potentially reflecting the earlier and more unstable stages of atherosclerotic mineralization. Here, we have confirmed that increased ^18^F-fluoride uptake provides an assessment of calcification activity within the coronary arteries, with uptake predicting progression in macroscopic calcium measured in individual plaques on CT (Figure 1), similar to previous results in the aortic valve and mitral valve annulus.^28,29^ Indeed, while calcium scores remained static in lesions without uptake, there was progression of calcification in plaques with increased ^18^F-fluoride uptake, with an ≈50% increase in median calcium score and calcium mass in as little as 12 months. Furthermore, the median change in calcium score in individual lesions with ^18^F-fluoride uptake was almost 20× greater than the change in calcium score in those without uptake. In individual coronary lesions, this finding remained even following adjustment for baseline calcium mass.

While the temporal nature of ^18^F-fluoride activity remains unknown, it is possible that ^18^F-fluoride uptake may increase as the atheromatous plaque begins to stabilize. Previous reports have highlighted that, while coronary ^18^F-fluoride uptake can be localized to culprit plaques in the majority of patients following acute myocardial infarction, quantitative PET activity, as measured by tissue-to-background ratio, is higher in patients with stable coronary artery disease compared with immediately following acute myocardial infarction.^11^ In a recent post hoc analysis of nearly 300 patients with established coronary artery disease, increased ^18^F-fluoride activity was an independent predictor of fatal and nonfatal myocardial infarction. Quantification of coronary microcalcification activity, a novel marker of ^18^F-fluoride uptake, demonstrated powerful prediction of future adverse events leading to a 7-fold increased risk of fatal or nonfatal myocardial infarction in participants with elevated coronary microcalcification activity.^30^

In the present study, we have for the first time linked coronary ^18^F-fluoride activity to progression in coronary atherosclerotic calcification. The main hypothesis for this study was to investigate the relationship between ^18^F-fluoride activity and calcification progression. However, we also investigated the relationship between PET activity and other morphological features of atherosclerosis, including low attenuation plaque, noncalcified plaque and total plaque volume. We have demonstrated that total plaque volume and noncalcified plaque volume are higher in patients with increased ^18^F-fluoride activity. The presence of increased ^18^F-fluoride activity was also associated with a greater change in total calcified plaque volume.

The study highlights the high frequency of increased ^18^F-fluoride activity in patients with established multivessel coronary artery disease, with two-thirds of patients having evidence of increased activity in at least one vessel. The prevalence of increased ^18^F-fluoride uptake is similar to previous reports in clinically stable patients.^11,31^ Previous studies have reported that over one third of patients with calcium scores greater than 1000 do not have evidence of increased ^18^F-fluoride activity.^31^ In this study, we have shown that the majority of patients (92%) with calcium scores greater than 1000 had evidence of increased ^18^F-fluoride uptake in at least one vessel, suggesting ongoing calcification activity. Indeed, an increasing disease burden was associated with an increase in the proportion of patients with evidence of increased PET activity in at least one vessel. This may reflect the underlying study population whom were largely high risk, with the majority (70%) having prior acute coronary syndrome and over 80% having previous coronary revascularization.

We recognize that there are some limitations to our study. PET-CT imaging was performed in a single center with experience in coronary PET-CT, and we need to explore the generalizability of our findings across multiple centers with different scanners. The high prevalence of previous percutaneous coronary intervention and stenting precluded calcium scoring assessment from at least one arterial segment in many patients. We mitigated for this by excluding those participants who underwent revascularization in a previously untreated arterial segment before the follow-up scan, ensuring the calcium burden on the follow-up scan was not underestimated. Although scan-rescan reproducibility of CT calcium mass and volume within individual plaques has not been reported, repeatability and variability of CT calcium scoring protocols has been demonstrated to be <20%.^32^ Furthermore, our scans were all performed on a single imaging system with the same scanning protocol and reconstruction protocol, thereby minimizing interscan variability. Lastly, our population represents a high-risk patient group with advanced disease, and the majority having had a previous acute coronary event. Hence, these results may not be applicable to all patients with known or suspected coronary disease and future studies in different risk groups would be welcomed. While recent data have highlighted the predictive value of ^18^F-fluoride activity in cardiovascular risk stratification, ultimately, prospective studies investigating the relationship between coronary ^18^F-fluoride uptake and cardiovascular events will determine the prognostic utility of this novel imaging method and observational studies in this field are ongoing (PREFFIR, URL: https://www.clinicaltrials.gov; Unique identifier: NCT02278211).

In conclusion, increased coronary ^18^F-fluoride uptake is associated with more rapid progression of coronary calcification at one year in patients with clinically stable multivessel coronary artery disease. In individual diseased segments, this association is independent of baseline calcium score, but at the patient level this finding is not independent of baseline calcium burden. ^18^F-fluoride uptake provides new insights into disease activity and progression of coronary atherosclerosis.

## Sources of Funding

This study was funded by a Wellcome Trust Senior Investigator Award (WT103782AIA) and an unrestricted educational grant from AstraZeneca. Dr Adamson is supported by a Heart Foundation of New Zealand Senior Fellowship (1844). Drs Doris, Dweck, and Newby are supported by the British Heart Foundation (FS/17/79/33226, FS/14/78/31020, CH/09/002, RG/16/10/32375, RE/18/5/34216). Dr Newby is a recipient of a Wellcome Trust Senior Investigator Award (WT103782AIA). Dr Moss is supported by a British Heart Foundation Accelerator Award Clinical Lectureship (AA/18/3/34220). E.J.R. van Beek is supported by the Scottish Imaging Network: A Platform of Scientific Excellence (SINAPSE). The Edinburgh Clinical Research Facilities and Edinburgh Imaging facility is supported by the National Health Service Research Scotland (NRS) through National Health Service Lothian Health Board. The DIAMOND (Dual Antiplatelet Therapy to Reduce Myocardial Injury) investigators acknowledge the contributions of the independent members of the trial steering committee; Professor Martin Wilkins, Professor Reza Razavi, Professor Robert F. Storey, Dr Dev Churamani, Chris Coner, and Rod Mycock. The authors acknowledge the contributions of Audrey Kuchnowski, Edwin Carter, and staff at the Wellcome Trust Clinical Research Facility, Edinburgh Clinical Trials Unit and Edinburgh Imaging Facility at the Royal Infirmary of Edinburgh.

## Disclosures

Dr Newby has received educational grants, honoraria for consultancy, and lectures from AstraZeneca. The other authors report no conflicts.

## Supplementary Material


