# Supplementary material for: Coronary 18F-Fluoride Uptake and Progression of Coronary Artery Calcification
Source: Circ Cardiovasc Imaging. 2020 Dec 15;13(12):e011438. doi: 10.1161/CIRCIMAGING.120.011438 (PMC7771641; doi:10.1161/CIRCIMAGING.120.011438)

SUPPLEMENTAL MATERIAL

Supplemental Table 1. Inclusion and exclusion criteria.

**Inclusion Criteria**
For inclusion in the study subjects should fulfil the following criteria:<br>1. Patients aged ≥40 years with angiographically proven multivessel coronary artery disease defined as at least two major epicardial vessels with any combination of either (a) >50% luminal stenosis, or (b) previous revascularization (percutaneous coronary intervention or coronary artery bypass graft surgery).<br>2. Provision of informed consent prior to any study specific procedures<br>3. Receiving aspirin
**Exclusion Criteria**
Subjects should not enter the study if any of the following exclusion criteria are fulfilled:<br>1. An acute coronary syndrome within the last 12 months<br>2. An indication for dual anti-platelet therapy, such as drug eluting stent<br>3. Receiving thienopyridine therapy such as clopidogrel or prasugrel<br>4. Percutaneous coronary intervention or coronary artery bypass graft surgery within the last 3 months<br>5. Inability or unwilling to give informed consent<br>6. Women who are pregnant, breastfeeding or of child-bearing potential (women who have experienced menarche, are pre-menopausal and have not been sterilised) will not be enrolled into the trial<br>7. Known hypersensitivity to ticagrelor or one of its excipients<br>8. Active pathological bleeding or bleeding diathesis<br>9. Significant thrombocytopenia: platelets <100 x 10^9 /L<br>10. History of intracranial haemorrhage<br>11. Moderate to severe liver impairments (Child's Grade B or C)<br>12. Maintenance therapy with strong CYP3A4 inhibitors, such as ketoconazole, nefazodone, ritonavir, indinavir, atazanavir, or clarithromycin<br>13. Major intercurrent illness of life expectancy <1 year

14. Renal dysfunction (eGFR ≤30 mL/min/1.73m^2)
15. Contraindication to iodinated contrast agents
16. Planned coronary revascularization or major non-cardiac surgery in the next 12 months
17. Maintenance therapy with simvastatin or lovastatin at doses greater than 40mg daily
18. Receiving oral anticoagulants including warfarin, rivaroxaban, dabigatran or apixaban

Supplemental Table 2. Calcium mass score calibration table. Minimum and maximum equivalent water diameter (Dw) values were calculated from CT images of a phantom with and without an obese expansion ring. Body mass index (BMI) and lateral diameter were calculated from Dw as previously described (15). Linear interpolation of calibration factor and anthropomorphic measurements was used between maximum and minimum values.

Equivalent water diameter<br>Dw (mm)	Body-mass index	Lateral dimension (coronal)<br>(mm)	Calibration factor at specified tube kilovoltage<br>120 kV
205	8.5	324	0.777
210	9.8	332	0.780
215	11.0	340	0.783
220	12.2	349	0.786
225	13.4	357	0.789
230	14.6	365	0.792
235	15.9	373	0.795
240	17.1	381	0.798
240	17.1	381	0.798
245	18.3	390	0.801
250	19.5	398	0.804
255	20.7	406	0.807
260	22.0	414	0.810
265	23.2	422	0.813
270	24.4	431	0.816
275	25.6	439	0.819
280	26.8	447	0.822
285	28.0	455	0.825
290	29.3	463	0.828
295	30.5	472	0.831
300	31.7	480	0.834
305	32.9	488	0.837
310	34.1	496	0.840
315	35.4	504	0.843
317	35.9	508	0.844

Supplemental Table 3. CT-defined plaque characteristics in PET-positive and PET-negative participants

Plaque Characteristic	Overall (N=183)	PET positive (n=116)	PET negative (n=67)
Number of positively remodelled plaques			
0	112 (61%)	70 (60%)	42 (63%)
1	41 (22%)	25 (22%)	16 (24%)
2	23 (13%)	14 (12%)	9 (13%)
3	5 (2.7%)	5 (4.3%)	0 (0%)
4	1 (0.5%)	1 (0.9%)	0 (0%)
5+	1 (0.5%)	1 (0.9%)	0 (0%)
Number of low attenuation plaques			
0	164 (90%)	104 (90%)	60 (90%)
1	17 (9.3%)	10 (8.6%)	7 (10%)
2	2 (1.1%)	2 (1.7%)	0 (0%)
Number of plaques with the napkin ring sign	4 (2.2%)	3 (2.6%)	1 (1.5%)
Number of plaques with spotty calcification			
0	116 (63%)	79 (68%)	37 (55%)
1	34 (19%)	21 (18%)	13 (19%)
2	16 (8.7%)	8 (6.9%)	8 (12%)
3	12 (6.6%)	4 (3.4%)	8 (12%)
4	4 (2.2%)	4 (3.4%)	0 (0%)
5+	1 (0.5%)	0 (0%)	1 (0.5%)
Number of plaques with punctate calcification			
0	179 (98%)	114 (98%)	65 (97%)
1	2 (1.1%)	0 (0%)	2 (3.0%)
2	2 (1.1%)	2 (1.7%)	0 (0%)
Total number of HRP per patient	1.00 [0.00-3.00]	1.00 [0.00-2.25]	1.00 [0.00-3.00]

Data presented as n (%) and median [IQR]

Supplemental Table 4. Average calcium density in PET-positive and PET-negative segments.

	**All patients (n=110)**	**PET positive (n=110)**	**PET negative (N=110)**	**P value**
**BASELINE**				
**Average Density (mg/mm^3)**	0.20 [0.18-0.23]	0.20 [0.16-0.23]	0.19 [0.16-0.22]	0.15
**FOLLOW-UP**				
**Average Density (mg/mm^3)**	0.21 [0.18-0.23]	0.21 [0.18-0.23]	0.19 [0.17-0.23]	**0.046**
**PROGRESSION**				
**Change in Average Density (mg/mm^3)**	0.009 [0.002-0.018]	0.01 [-0.003-0.03]	0.01 [-0.01-0.03]	0.102

Supplemental Figure 1. An example of per-segment PET-CTA and CT calcium scoring image analysis. In this participant, the PET-positive segment was selected as the proximal right coronary artery which demonstrated increased ^{18}F-fluoride activity (A, red arrow). At baseline, the calcium score of this segment was 496 AU (B) and subsequently increased to 944 AU at 12 months. In this participant, the proximal left circumflex artery was selected as the PET-negative reference lesion (C, blue arrow). At baseline, the calcium score in this segment was 496AU and subsequently increased to 616 at 12 months.

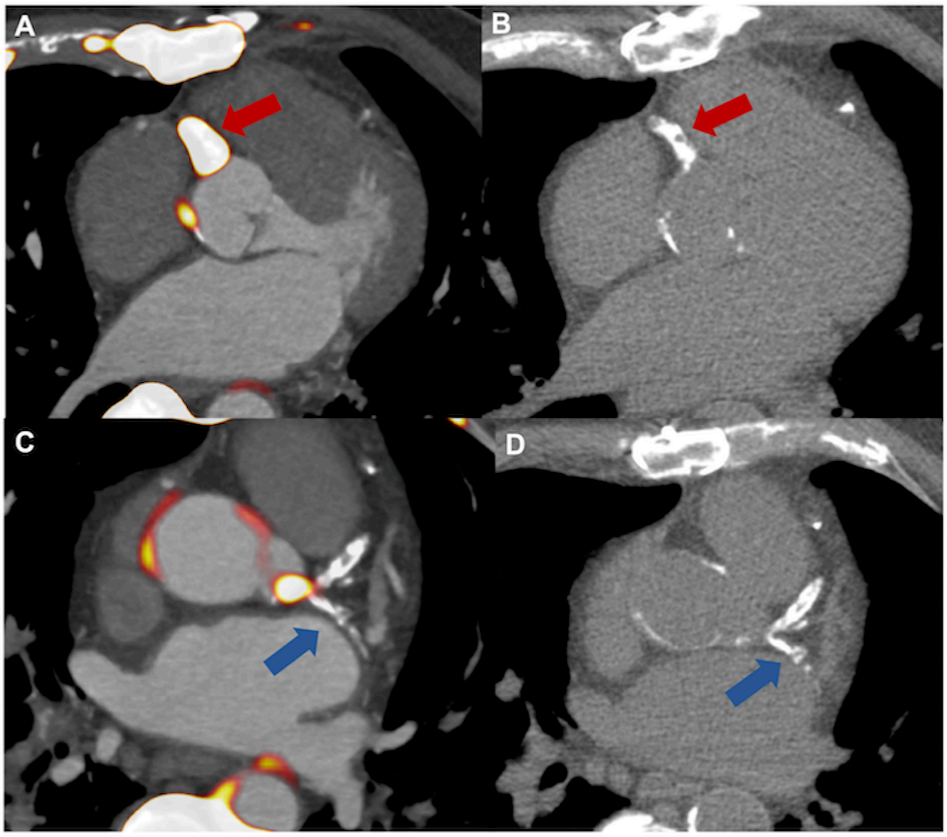

Supplemental Figure 2. Consort Diagram.

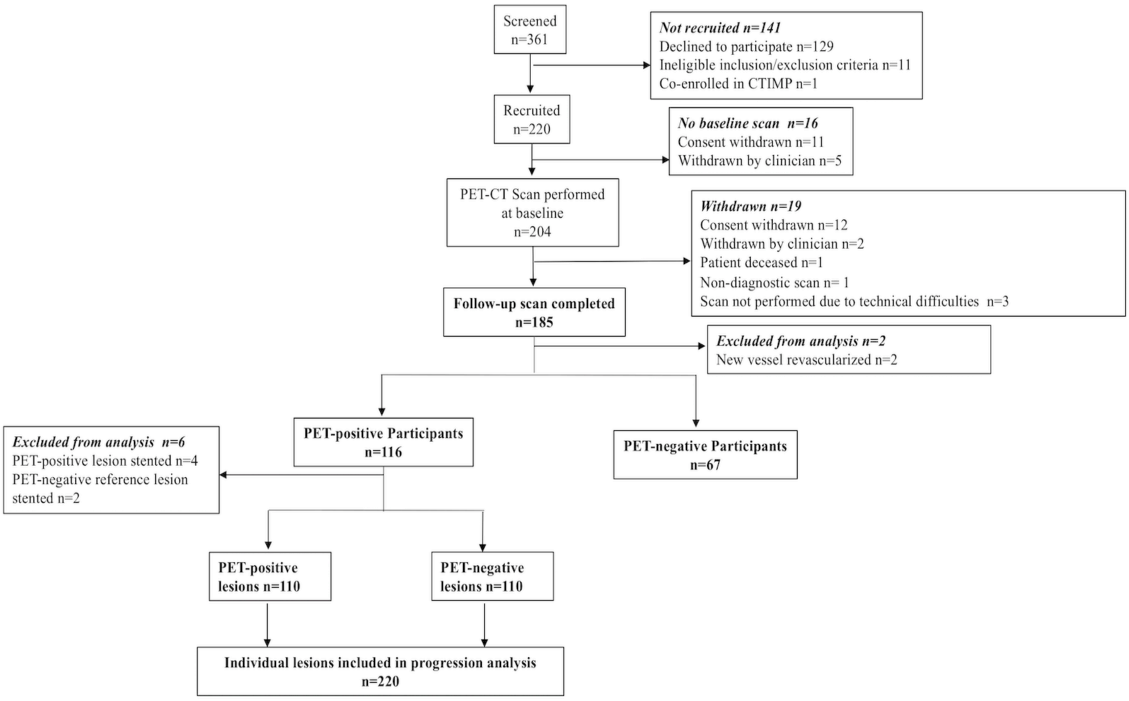

Supplement: Supplementary file 1 [file hci-13-e011438-s001.pdf]
